# Final planned overall survival (OS) from OPTiM, a randomized Phase III trial of talimogene laherparepvec (T-VEC) versus GM-CSF for the treatment of unresected stage IIIB/C/IV melanoma (NCT00769704)

**DOI:** 10.1186/2051-1426-2-S3-P263

**Published:** 2014-11-06

**Authors:** Robert HI Andtbacka, Frances A Collichio, Thomas Amatruda, Neil Senzer, Jason Chesney, Keith Delman, Lynn Spitler, Igor Puzanov, Sanjiv Agarwala, Mohammed Milhem, Kevin Harrington, Mark Middleton, Ai Li, Mark Shilkrut, Robert Coffin, Howard Kaufman

**Affiliations:** 1 Huntsman Cancer Institute, Salt Lake City, UT, USA; 2 The University of Carolina at Chapel Hill, Chapel Hill, NC, USA; 3 Minnesota Oncology, MN, USA; 4 Mary Crowley Cancer Research Center, TX, USA; 5 University of Louisville, Louisville, KY, USA; 6 Emory University, Atlanta, GA, USA; 7 Northern California Melanoma Center, San Francisco, CA, USA; 8 Vanderbilt University Medical Center, Nashville, TN, USA; 9 St. Luke's University Hospital & Health Network, Bethlehem, PA, USA; 10 University of Iowa, Iowa City, IA, USA; 11 The Institute of Cancer Research/The Royal Marsden Hospital, London, UK; 12 National Institute for Health Research Biomedical Research Centre, London, UK; 13 Amgen Inc, Thousand Oaks, CA, USA; 14 Amgen, Woburn, MA, USA; 15 Rutgers Cancer Institute of New Jersey, New Brunswick, NJ, USA

## Background

T-VEC is an oncolytic immunotherapy derived from herpes simplex virus type-1 designed to selectively replicate within tumors and to produce GM-CSF to enhance systemic antitumor immune responses. OPTiM, a randomized Phase III trial of T-VEC vs GM-CSF in patients with unresected melanoma with regional or distant metastases met the primary objective of an improvement in durable response rate (response lasting continuously for ≥6 months) with T-VEC versus GM-CSF (16% vs 2%, respectively; *P*<0.001). Most common adverse events with T-VEC were fatigue, chills, and pyrexia. No ≥grade 3 adverse events occurred in ≥3% of patients in either arm (Andtbacka et al., *J Clin Oncol *2013,32[suppl]:LBA9008). At the primary analysis (PA) of secondary OS endpoint, with median follow-up of 44 (range, 32-59) months and 189 events in the T-VEC arm and 101 events in the GM-CSF arm, median (95%CI) OS was 23.3 (19.5-29.6) months for T-VEC and 18.9 (16.0-23.7) months for GM-CSF (hazard ratio [HR]=0.79; 95%CI = 0.62-1.00; *P *= 0.051) (Kaufman et al., *J Clin Oncol *2014,32[suppl]:9008a). A planned analysis of OS at 3 years from the last randomization is presented here.

## Methods

Eligible patients were ≥18 years old; had ECOG performance status (PS) ≤1; unresectable melanoma stage IIIB/C/IV; injectable cutaneous, subcutaneous (SC) or nodal lesions; LDH≤1.5X upper limit of normal; ≤3 visceral lesions (excluding lung), none>3 cm. Patients were randomized 2:1 to intralesional T-VEC (initially ≤4 mL x10^6 ^pfu/mL, then after 3 wks, ≤4 mL x10^8 ^pfu/mL q2w) or SC GM-CSF (125 µg/m^2^qd × 14 ds q4w).

## Results

Of 436 patients in the intent-to-treat analysis, 295 (68%) patients received T-VEC and 141 (32%) patients received GM-CSF; 57% were men; median age 63 yrs. At time of the final OS analysis with median follow-up of 49 months [range, 37-63], only 1 additional event occurred (T-VEC arm). Median (95%CI) OS was 23.3 months (95%CI = 19.5-29.6) for T-VEC and 18.9 months (16.0-23.8) for GM-CSF; HR = 0.80 (95%CI = 0.62-1.01), *P *= 0.06 (descriptive). Five-year survival for the T-VEC arm was 33.4% (95%CI = 27.7-39.2). T-VEC effect on OS was most pronounced in patients with stage IIIB/C/IVM1a melanoma (HR = 0.57; 95%CI = 0.41-0.81, *P *= 0.001 [descriptive]) and in patients with treatment-naive disease (HR = 0.52; 95%CI = 0.36-0.75, *P *< 0.001 [descriptive]).

## Conclusions

With >4 years of median follow-up for survival, a persistent relevant OS effect was demonstrated with further follow-up. Long-term follow-up continues in the registry trial (NCT02173171). T-VEC represents a novel potential therapy for patients with regionally and distantly metastatic melanoma.

